# Age and sex dependent changes in liver gene expression during the life cycle of the rat

**DOI:** 10.1186/1471-2164-11-675

**Published:** 2010-11-30

**Authors:** Joshua C Kwekel, Varsha G Desai, Carrie L Moland, William S Branham, James C Fuscoe

**Affiliations:** 1 Center for Functional Genomics, Division of Systems Biology, National Center for Toxicological Research, U.S. Food and Drug Administration, 3900 NCTR Road, Jefferson, Arkansas 72079, USA

## Abstract

**Background:**

Age- and sex-related susceptibility to adverse drug reactions and disease is a key concern in understanding drug safety and disease progression. We hypothesize that the underlying suite of hepatic genes expressed at various life cycle stages will impact susceptibility to adverse drug reactions. Understanding the basal liver gene expression patterns is a necessary first step in addressing this hypothesis and will inform our assessments of adverse drug reactions as the liver plays a central role in drug metabolism and biotransformation. Untreated male and female F344 rats were sacrificed at 2, 5, 6, 8, 15, 21, 52, 78, and 104 weeks of age. Liver tissues were collected for histology and gene expression analysis. Whole-genome rat microarrays were used to query global expression profiles.

**Results:**

An initial list of differentially expressed genes was selected using criteria based upon p-value (p < 0.05) and fold-change (+/- 1.5). Three dimensional principal component analyses revealed differences between males and females beginning at 2 weeks with more divergent profiles beginning at 5 weeks. The greatest sex-differences were observed between 8 and 52 weeks before converging again at 104 weeks. K-means clustering identified groups of genes that displayed age-related patterns of expression. Various adult aging-related clusters represented gene pathways related to xenobiotic metabolism, DNA damage repair, and oxidative stress.

**Conclusions:**

These results suggest an underlying role for genes in specific clusters in potentiating age- and sex-related differences in susceptibility to adverse health effects. Furthermore, such a comprehensive picture of life cycle changes in gene expression deepens our understanding and informs the utility of liver gene expression biomarkers.

## Background

The liver's ability to process exogenous chemicals and protect itself from injury is influenced by many factors, including an individual's age [1], sex [2], ethnicity [3], genotype [4], and diet [5]. Age- and sex-dependent factors are decidedly innate and comprise two of the most important variables in gauging liver function and responsiveness to chemical insult. Because the liver is the first and primary line of defense against the potentially toxic effects of oral xenobiotics [6,7], inquiry into its gene expression profiles to assess potential susceptibilities was performed. Not all potential causes of susceptibility were examined, only those gene expression changes associated with sex and age. Some important gene regulatory events may not be evident at the RNA level, in the tissue examined (liver), or in the absence of chemical challenge. Mechanisms of susceptibility may also be dependent on non-transcriptional events such as protein expression and modifications, as well as polymorphisms in specific genes and proteins, which lie beyond the scope of the current study. Nonetheless, capturing baseline gene expression profile measurements is a necessary first step in characterizing the suite of genes differentially expressed at various ages.

Age-dependency in susceptibility to drug induced liver injury (DILI) has been established clinically. For example, age has been shown as a predisposing factor for adverse effects of the antiepileptic drugs carbamazepine and valproate [8]. In the case of carbamazepine, elderly patients appear to be at increased risk of developing blood dyscrasias and liver reactions whereas risk for adverse liver reactions to valproate is associated with young patients. Sex-differences in DILI have also been reported: for example, acetaminophen seems to exhibit female-specific susceptibilities in clinical and epidemiology studies [9,10]. However, it remains to be seen if higher rates of female DILI are due to intrinsic (biology) versus extrinsic (increased use) factors. Whether susceptibilities to DILI are generally more common in males versus females have not been as clearly demonstrated clinically when considering all drugs [11-13]. This suggests that when sex-specific susceptibilities are present, the sex-specificity varies from drug to drug.

It is well understood that the liver's drug metabolizing capacity is a function of its gene expression levels [14]. Furthermore, transcriptome profiling continues to play an increasing role in estimating toxicity and efficacy of various drugs in the liver in preclinical and clinical evaluations [15,16]. Several prior studies have evaluated hepatic gene expression differences between young and old animals or evaluated sex differences on a relatively limited scale [17-22]. However, a more comprehensive evaluation of the whole genome during time points spanning immature, pubertal, early adult and aging adult animals, in both sexes, would provide greater interpretive power to assess life cycle liver gene expression and its impact on drug safety and disease. This study provides a comprehensive look at liver gene expression in untreated male and female rats throughout the entire life cycle in order to assess the basal level profiles of liver genes that may underlie patterns of susceptibility due to sex and age.

## Results

**Figure 1:**
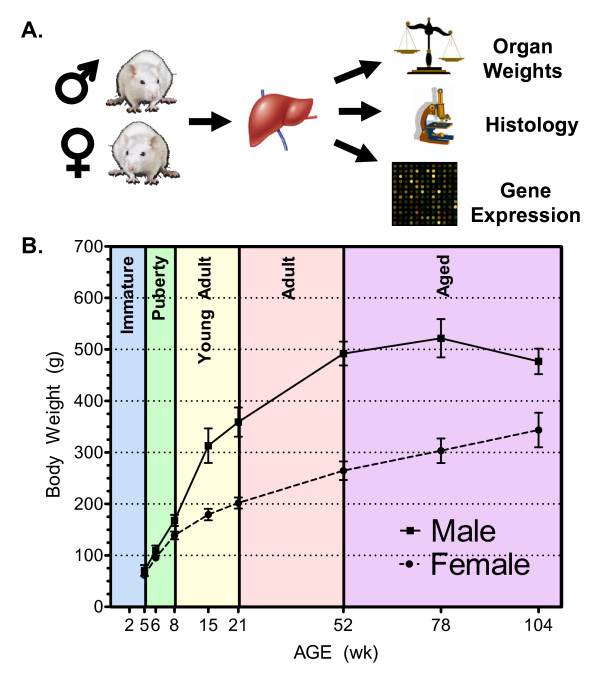
**Rat life cycle study design and animal body weight growth curves**. Untreated male and female F344 rats (A.) were sacrificed at 2, 5, 6, 8, 15, 21 weeks (male and female n = 6 per age group), 52 weeks (males n = 10, female n = 10), 78 weeks (male n = 8, female n = 8) and 104 weeks (male n = 6, female n = 16) of age corresponding to essential life stages (B.) during animal growth and maturation. Liver tissues were collected and used for whole rat genome expression profiling (n = 5 per sex, per age group). Animal body weights (n = 6 to 16, +/-SEM) were recorded at necropsy. Two week old animals were not weighed to decrease handling stress. Rapid growth during early development subsides after 15 weeks and males reach nearly twice the weight of females at 52 weeks.

A comprehensive time-course study, comprising nine age groups spanning the entire life cycle (between 2 weeks and 2 years of age) of Fischer 344 rats was performed in both male and female animals (Figure 1A). Terminal body weights were recorded for each animal (Figure 1B) and the corresponding growth curves are consistent with historical findings [23]. An original number of sixteen animals of both sexes was included in the 104 week group. However, consistent with previous reports [24], 10 male animals were found to be moribund or died prior to 104 weeks and were thus removed from the study. No female animals were removed early. Pathological examinations were performed on animals at 52, 78 and 104 weeks of age. Neoplastic histopathological findings in the liver which showed sex-related trends included a hepatocellular adenoma in only one 104 week male. Non-neoplastic findings included basophilic foci in females (3 and 12 animals at 78 and 104 weeks, respectively) but not in males. Also, male predominant findings of bile duct hyperplasia with increasing incidence with age were observed. In summary, the incidences of neoplastic and non-neoplastic lesions were typical of those observed in historical control Fischer 344 rats of comparable age [25].

**Figure 2:**
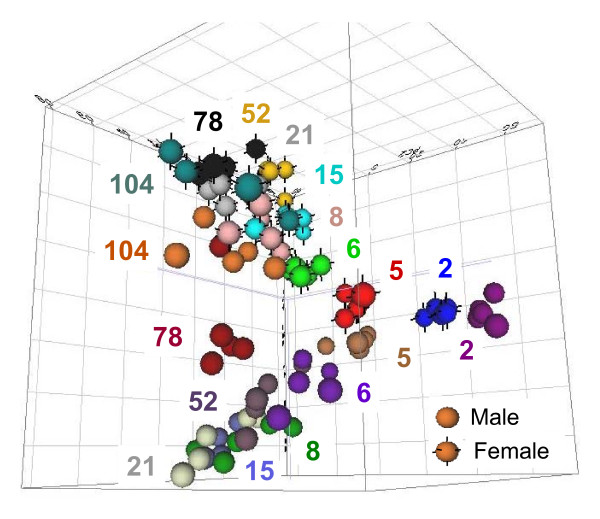
**Three-dimensional principal component analysis**. Global relationships between the expression patterns of individual animals were visualized using the 7,951 differentially expressed features meeting the initial filtering criteria (p < 0.05 and at least +/- 1.5 relative fold change). Each sphere represents the expression from one animal. Animals of the same sex and age have the same color and are labelled by age in weeks (ArrayTrack). Females are indicated by black vertices or whiskers, males have none. Together, these data illustrate the relatively high reproducibility between biological replicates (n = 5) in a discrete and continuous linear pattern from young to old animals. It also suggests clear sex- and age-dependent differences in liver gene expression, with males and females exhibiting diverging profiles beginning at 2 weeks with greatest differences observed at 21 and 52 weeks before converging again at 104 weeks.

Whole genome profiling of rat liver gene expression was performed (n = 5) in each sex and age group using Agilent microarrays. These rat whole genome microarrays contain 43,379 features or spots of which 23,233 have Entrez Gene IDs, 16,327 of which are unique. Gene expression data were entered into ArrayTrack™ [26], the Food and Drug Administration's database for microarray data storage, processing, analysis and visualization that was created at the National Center for Toxicological Research (NCTR). The composite liver gene expression profiles for all genes (unfiltered) displayed good reproducibility in general between animals of the same sex and age group with average Pearson's correlations of R = 0.986 between biological replicates of the same sex and age. Normalized intensity values were analyzed using a two-way ANOVA (sex, age) to calculate a p-value for statistical evaluation. Data for each spot across all arrays were mean-scaled to calculate relative fold-change differences in expression for each sex and age. The initial filtering criteria of p < 0.05 for sex or age difference with an absolute fold-change greater than or equal to 1.5 in relative expression at any age was used to define a set of 7,951 differentially expressed features comprising 3,770 unique Entrez Gene IDs or differentially expressed genes (DEGs). Using these 7,951 differentially expressed features, global expression pattern analysis was performed using three-dimensional principal component analyses (3D-PCA) within ArrayTrack, wherein the three principal components capturing the highest amount of variability in the DEG data were plotted (Figure 2). These three principal components captured approximately 58% of the total variability present in the data (31.4%, 16.2% and 10.2% for PC1, PC2 and PC3, respectively). Three-dimensional PCA facilitates the visualization of individual animal expression data within each sex and age group and the global relationships between one another. Male and female animals showed clear separation from each other for most of the life cycle. Furthermore, most, if not all age groups displayed temporal continuity with neighboring age groups in a contiguous manner (i.e., expression profiles from 6 week old animals of each sex lay in-between those of 5 week and 8 week animals in 3D-space, etc.). Lastly, the largest sex-differences in expression occurred between 15 and 52 weeks of age, whereas the biggest age-related differences occurred, as expected, between the youngest animals (2 weeks) and adult animals (52 weeks).

**Figure 3:**
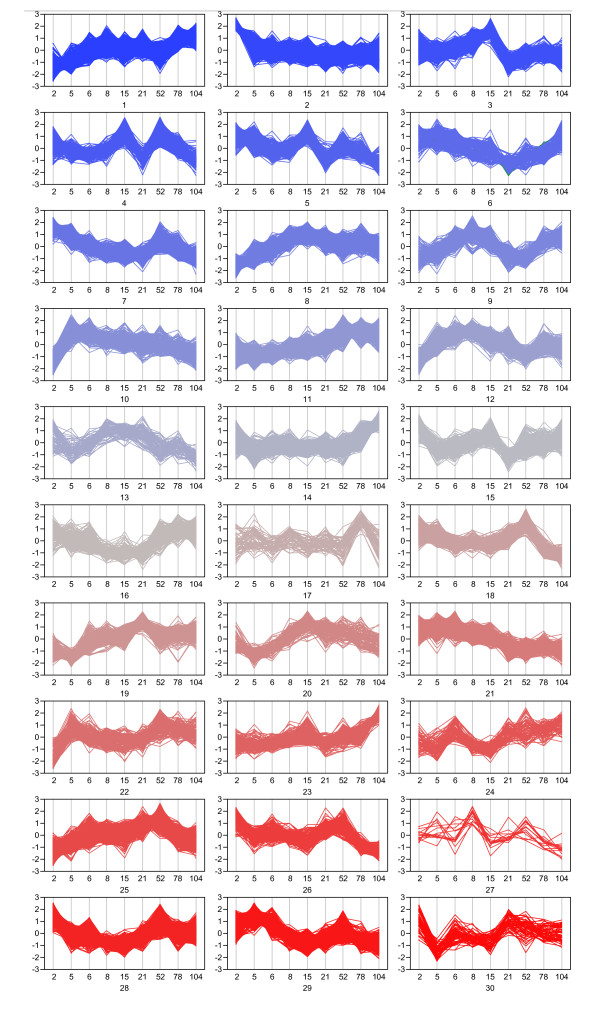
**K-means cluster analysis of differentially expressed genes**. Differentially expressed genes were clustered into 30 k-means clusters (JMP Genomics, SAS 9.2) as this is the lowest number of clusters which allows a minimum correlation coefficient of R = 0.7 between any one expression profile and its other cluster members. Male and female data were allowed to cluster independently between the 30 groups such that male and female data might cluster to separate groups for a given gene. The x- and y-axes represent age and relative fold change, respectively. Color is arbitrary and does not represent sex. These data illustrate the various biological patterns of liver expression which exist during pre-pubertal, pubertal, early adult and aged rat life stages.

A second clustering method, k-means clustering (JMP Genomics 4.0, SAS. Cary, NC), was performed to show individual gene profiles and to find groups of genes with similar patterns of expression associated with specific stages of development or aging (Figure 3). Male and female data for each array feature were allowed to cluster independently such that male and female data for a given gene may group into separate clusters. The number of clusters was determined empirically according to the fewest clusters required to achieve a minimum correlation radius of 0.7 between any individual profile and its cognate cluster members, resulting in 30 clusters. K-means cluster analysis allowed for grouping of genes based upon similarity of expression profiles across all ages. The number of features included in each cluster ranged from 16 to 2,252. Many clusters reflected temporal patterns that could be readily interpreted in the physiological context, such as high and low perinatal expression (clusters 2 and 10, respectively) or up- (clusters 6, 9, 14, 23) and down- (clusters 13, 18, 25, 26) regulation in the aging animals. Clusters also displayed both sustained (clusters 2, 10, 11, 21) and transient (clusters 3, 4, 15, 22, 24) patterns of regulation. Although not all of the clusters are readily interpretable, knowledge of rat growth patterns, sexual maturation, and basic liver function assist in interpreting time-course gene expression data. For example, cluster 2 and cluster 10 (Figure 3) are negatively correlated with each other yet both exhibit early and substantial changes (between 2 weeks and 5 weeks) followed by negligible change for the rest of the life cycle. Clusters 14 and 25 also negatively correlate with each other and show clear and consistent upward and downward trends, respectively, in the late age groups (between 52 weeks and 104 weeks). Thus, temporal patterns can be clearly distinguished for genes potentially involved in early development vs. late aging based upon these life cycle clusters.

**Figure 4:**
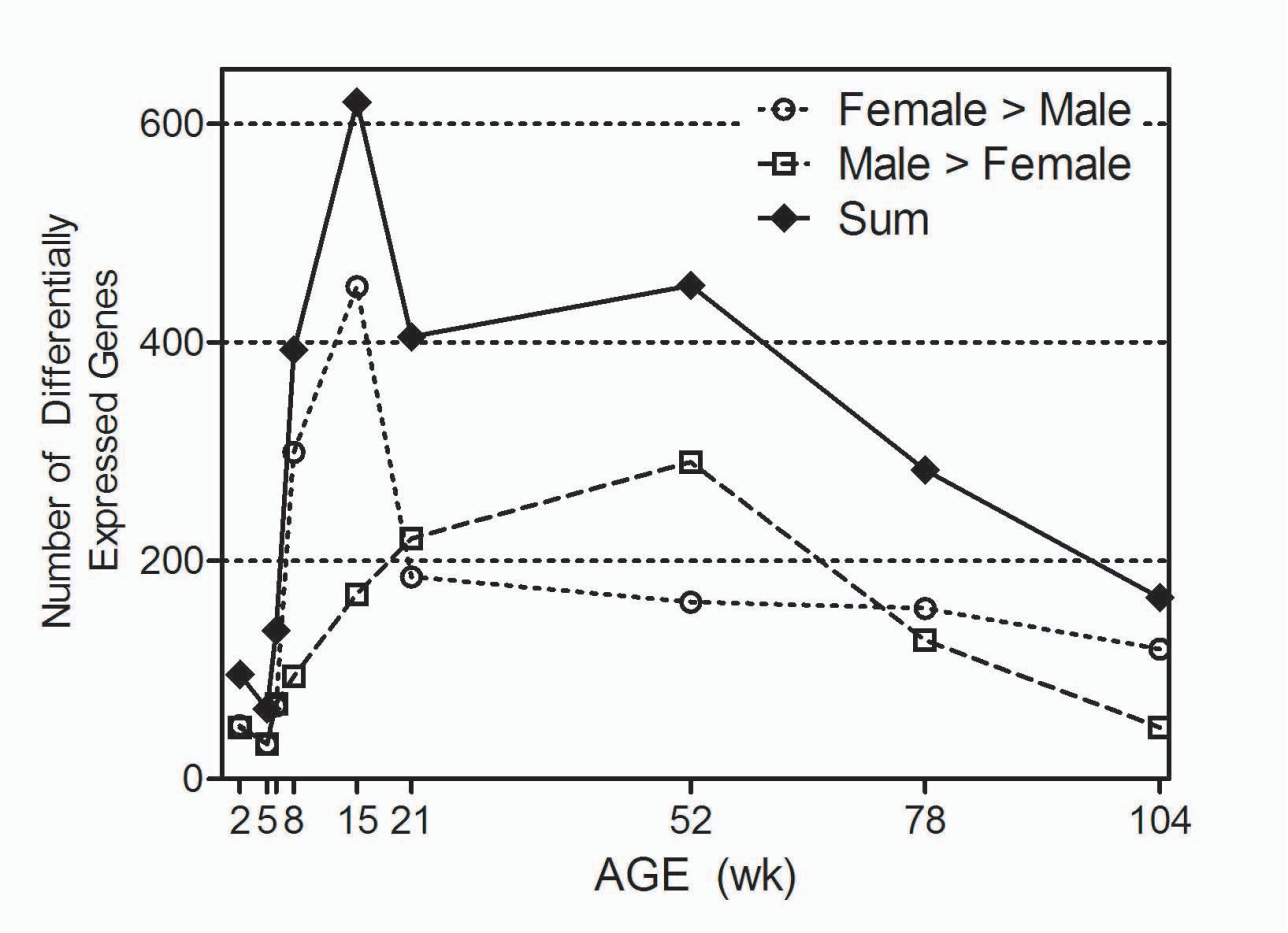
**Temporal profile of sex-differences in liver gene expression**. The number of genes exhibiting a minimum 2-fold difference in expression between male and female animals was calculated at each age group. These data illustrate increased female expression over males at 8 and 15 weeks which then is reversed during early adulthood (21 to 52 weeks) where males show a greater number of genes with higher gene expression than females.

Sets of genes for which expression was at least 2-fold greater in the opposite sex were determined for each age group (p-value < 0.05; fold-change > 2 between sexes) from the set of 3,770 DEGs described above. The numbers of these sex-predominant genes at each age are presented in Figure 4. The resulting plots indicate a notable period from 6 to 21 weeks of age in which more than 70% of the genes showing sex-predominance are expressed at a higher level in females than males. From the ages of 21 to 78 weeks, sex-predominant gene expression was higher in males than females.

An unsupervised, pathway-analysis approach was performed to identify functional categories of gene response. Analysis software Ingenuity Pathway Analysis (IPA) was used to assist in biological interpretation of the expression data from the initial list of 3,770 DEGs with unique EntrezGene IDs. Pathways which exhibited large numbers of DEGs included metabolism of xenobiotics by cytochrome P450, fatty acid metabolism, tryptophan metabolism, and acute phase response. Genes involved with liver cholestasis, liver damage and liver necrosis/cell death were highly represented in toxicity-related functional analysis of the DEGs. Furthermore, gene networks represented by the DEGs included seven high-scoring IPA categories, including cell mediated immune response, cell growth and proliferation, lipid metabolism, organ morphogenesis, cell movement, and hepatic system disease.

Since data from all age groups were included in the pathway analysis, the results are generic to the entire life cycle and do not provide meaningful insight into specific periods of the life cycle which may feature unique expression patterns that would underlie age-related susceptibilities. Thus, a more focused functional analysis of targeted age groups was implemented. Adult aging-related clusters (up-regulated with age: clusters 6, 14, 23; down-regulated with age: clusters 13, 18, 25, 26; Figure 3) from the k-means cluster analysis were selected and their corresponding gene lists were queried for pathways. Genes associated with organ injury/abnormality (Crp (C-reactive protein), Il6st (interleukin 6 signal transducer), Stat3 (signal transducer and activator of transcription 3)) and lipid metabolism (Pon1 (paraoxonase 1), Apoa1 (apolipoprotein A1), Cyp27a1, Abcg1 (ATP-binding cassette G1)), as well as toxicity-related functions of liver cholestasis, necrosis and cell death, were all up-regulated during the second year of the rat life cycle. These gene family results overlap with the top pathways represented in the up-late clusters including acute phase response signaling, complement system genes and xenobiotic metabolism. The clustering of up-regulated gene families related to immune response and cell death signaling pathways match well with a presumed accumulation of aging hepatocytes [27], the potential hepatic accumulation of fatty acids, and increased demand for metabolic capacity for lipids and xenobiotics which accumulate in the fat of aging animals [28]. Genes that clustered in a pattern of down-regulation in the adult aging rats are more difficult to interpret as loss of expression may itself be a product of the aging process [29]. However, a number of represented gene networks in the aging-related down-regulation clusters included cell signaling genes particularly associated with the nervous system. A major hub of many of the top represented gene networks in both the up- and down-regulated, aging-associated clusters was NF-κB (nuclear factor κB).

A supervised (pathway driven) approach was used to specifically query three general gene ontology (GO) areas of interest, namely xenobiotic metabolism, DNA damage repair, and oxidative stress-related genes (Table 1). These gene categories are hypothesized to play important roles in sex- and age-related susceptibility to adverse drug effects [18,30]. Of the 122 genes included in the xenobiotic metabolism gene list in the Ingenuity Knowledge Base, 61 were differentially expressed. These included Cyp2d4, the rat ortholog of human gene CYP2D6, which is speculated to metabolize up to 25% of commonly prescribed drugs [31]. Genes involved in DNA Damage Repair, derived from Ingenuity, were combined with the list by Wood et al. [32] to give 222 genes involved in DNA damage repair. Sixty-five of these genes (approximately 25%) were found to be differentially expressed in the liver. Oxidative Stress genes were defined by 68 genes included in "response to oxidative stress" (IPA) of which 23 genes were differentially expressed (Table 1).

**Table 1:** Supervised analysis of differentially expressed genes from specified pathways

							Cluster**	
Category	Pathway	Entrez GeneID	Gene Symbol	P-value* (AGE)	P-value* (SEX)	P-value* (AGE^SEX)	Female	Male
DNA Damage Repair	BER	304577	Ung	0.0026	0	0.2116	1	1
		25332	Mgmt	0.778	0	0.0006	23	11
	MMR	294252	Msh5	0.3511	0.0043	0.6288	9	23
	NER	298074	Xpa	0.0002	0	0.7036	11	24
		59102	Rpa2	0.5979	0.0042	0.0102	14	2
		81513	Lig1	0.459	0	0.0009	6	2
	Other	85472	Polg	0.0001	0.0022	0.1838	2	30
		59294	Pold1	0.3243	0	0	14	2
		316344	Rev1	0.0007	0	0.0009	15	2
		84490	Fen1	0.0339	0	0.001	6	2
		308755	Blm	0.0008	0	0.0021	7	2
		309595	Mdc1	0.8699	0	0.0564	29	29

Xenobiotic Metabolism	Phase 1	24300	Cyp2b1	0.1891	0	0.3345	12	10
		378476	Cyp2c11	0	0	0	10	25
		171521	Cyp2c13	0	0	0	29	10
		266682	Cyp3a2	0	0	0	2	29
		252931	Cyp3a18	0	0	0	21	25
		170509	Cyp3a62	0	0	0	23	11
		266674	Cyp4a8	0	0	0	1	25
	Phase 2	24422	Gsta2	0.0189	0	0	1	8
		24423	Gstm1	0	0	0	12	8
		24424	Gstm2	0	0	0	1	8
		24862	Ugt2b	0.3287	0.006	0.0082	9	25
	Phase 3	25303	Abcc2	0	0	0.2455	8	8
		140668	Abcc3	0	0	0	21	6
		83500	Slc22a8	0	0	0	5	13
		0	Slco1b3	0	0	0.0053	1	11
		117048	Cdh17	0	0	0	4	25
		25027	Slc16a1	0	0	0.0207	21	2
		295356	Slc16a4	0	0	0	21	25

Oxidative Stress Response	Nrf2	25315	Ephx1	0.0091	0	0.0458	1	1
	Pathway	25256	Fmo1	0.1169	0.0008	0	6	10
		113894	Sqstm1	0.1126	0	0.0394	1	1
		24451	Hmox1	0.1788	0	0.182	14	14
		29292	Ftl	0.5177	0	0.2465	12	12
		24786	Sod1	0.0001	0	0.3873	8	1
		24248	Cat	0.0035	0	0.0148	1	19
		58819	Txnrd1	0.7899	0.0001	0.5142	12	10
		291796	Usp14	0.0053	0	0.0001	21	29

*Two-way ANOVA results concerning AGE and SEX, n = 5, on normalized intensity values. "0" indicates p-values less than 1 × 10-6. **K-means cluster analysis of expression profiles, agglomerative clustering, (Figure 3)

**Figure 5:**
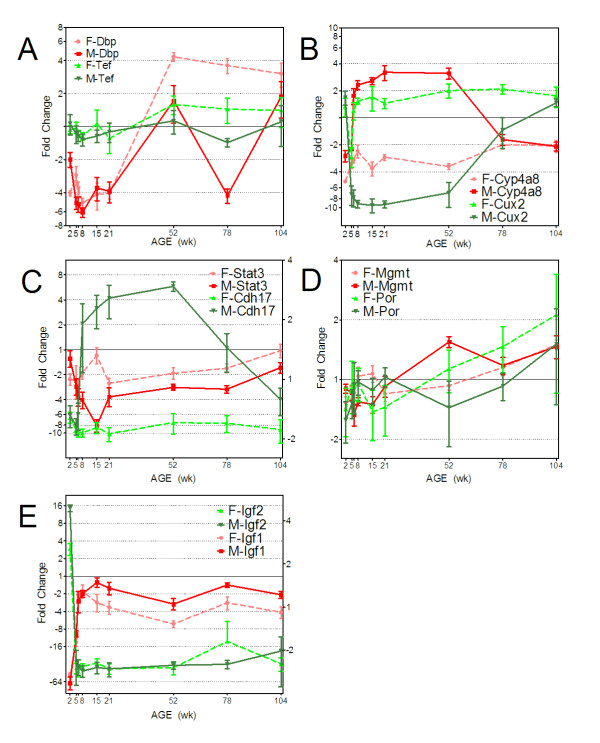
**Comparisons of individual expression profiles**. **(A.) **Transcription factors Dbp and Tef are controlled by the circadian clock-associated suprachiasmatic nucleus and exhibit conserved, sex-specific life cycle profiles. **(B.) **Cux2 functions as a female-specific repressor and putatively represses Cyp4a8 expression in females but not males in an age-dependent manner. **(C.) **Cdh17 exhibits male-specific predominance in expression from 8 weeks onward, while Stat3 shows female-specific predominance transiently at 15 weeks. **(D.) **Both Por and Mgmt exhibit trends of increasing expression during the second year of the rat life cycle and are involved in xenobiotic metabolism and DNA damage repair, respectively. **(E.) **Early developmental transition between Igf2-predominance at 2 weeks to Igf1-predominance by 6 and 8 weeks in the liver is shown at the expression level. The right Y-axes present in panels C and E represent data for Stat3 and Igf1, respectively, to show differences in scale.

**Figure 6:**
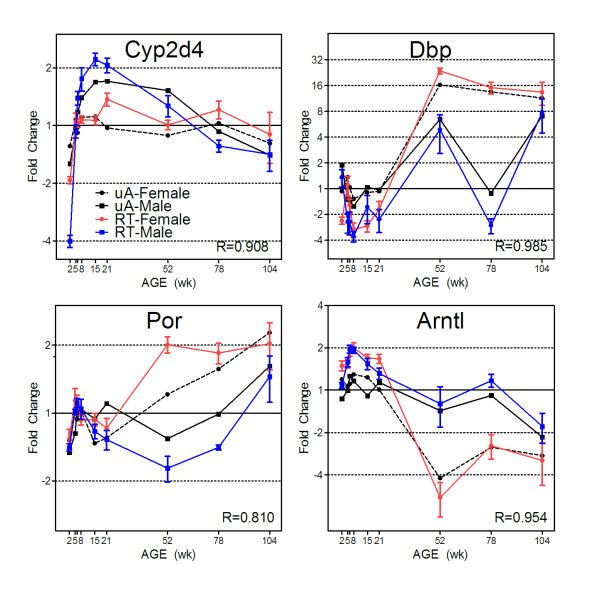
**Quantitative real time PCR verification of selected genes**. Microarray expression profiles for Cyp2d4, Por, Dbp and Arntl were assessed by qRTPCR using gene specific Taqman probes. Housekeeping gene beta-actin was used to standardize expression. In each case, the qRTPCR data very closely approximated the microarray expression levels and are temporally correlated in both sexes. Pearson's correlations were calculated between averaged microarray (uA) and qRTPCR (RT) data for each gene and coefficients ranged from R = 0.810 to 0.985. Blue and red lines indicate male and female qRTPCR data, respectively (n = 5), and black lines represent microarray data (n = 5, error bars represent SEM for qRTPCR).

**Figure 7:**
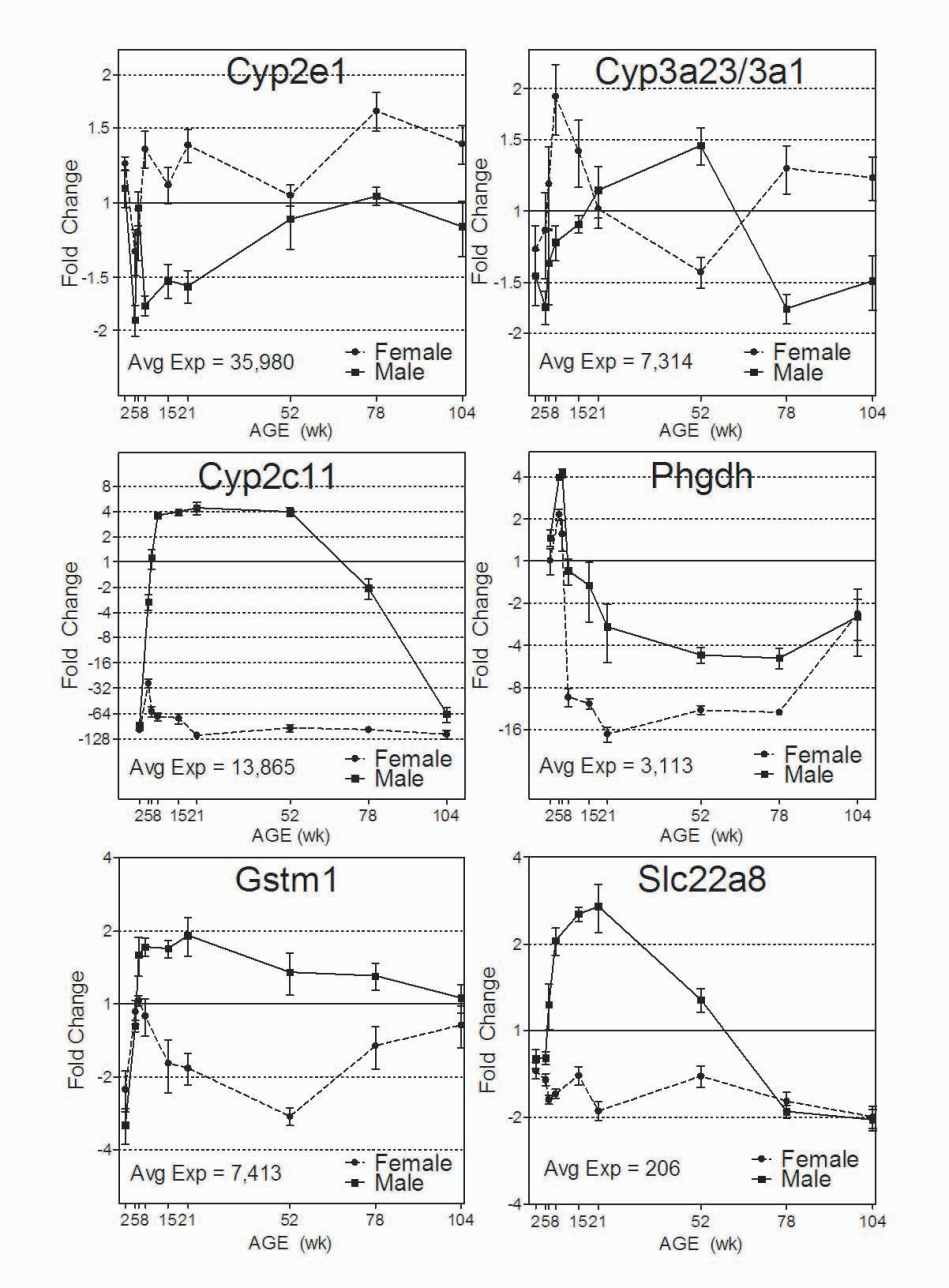
**Individual examples of expression profiles**. Microarray data fold-changes for selected genes are plotted (n = 5, error bars represent SEM). Solid and dashed lines indicate male and female expression, respectively. Averaged expression levels (normalized fluorescence intensities) across both sexes and all time points are reported for each gene and have been scaled to equal "1" in the graph.

As a result of the global cluster analysis methods, various gene relationships in the expression data were revealed from the analysis of correlated and anti-correlated expression profiles (Figure 5A and 5B). Examples of genes showing sex-divergent expression for both male and female specific regulation were identified (Figure 5C). Patterns of expression associated with specific developmental stages or periods of adult aging were also evident and suggest age-specific regulation in the liver (Figure 5D and 5E). Array based expression profiles were verified using quantitative real time PCR for four genes, using beta actin as a housekeeping control (Figure 6). Cyp2d4, the rat ortholog of human gene CYP2D6, and Por (P450 oxidoreductase) are important genes involved in Phase I xenobiotic metabolism. The other two genes (Dbp and Arntl) are involved in circadian rhythm regulation of gene expression in the liver. An average correlation of R = 0.914 between qPCR and array data was calculated for these four genes, demonstrating high agreement between the two methods. Expression profiles for specific examples of genes found in xenobiotic metabolism pathways (Cyp2c11, Cyp2e1, Cyp3a23/3a1, Gstm1, glutathione-s-transferase mu 1; Slc22a8, solute carrier family 22 member 8) and energy metabolism (Phgdh, 3-phosphoglycerate dehydrogenase), which exhibit notable sex and age related differences, are shown in Figure 7.

## Discussion

The assessment of gene expression changes at key developmental periods of the life cycle, as well as at the ages most commonly used in pre-clinical drug safety testing, was the focus of this study. Fischer 344 rats are frequently used in the National Toxicology Program and one of the most commonly used inbred strains for preclinical pharmaceutical in-life testing [25,33]. The two early time points, 2 and 5 weeks, span the time of weaning (3 weeks). The National Toxicology Program (NTP) 2-year bioassays are typically initiated in 6-8 week old rats [34], thus 6 and 8 week age groups were included to provide baseline for future NTP bioassays. The 15-week age group captures expression data after sexual and physical maturity [35]. Furthermore, a 13-week NTP sub-chronic study, initiated in 8 week old animals would be completed at 21 weeks. Reproductive senescence and aging phenotypes begin to be apparent during the second year [35], thus 52, 78, and 104 week age groups were assessed.

Data analysis began with an unsupervised, or data-driven, approach to interpreting the expression data. Clustering methods were used to describe the global relationships within the data on a per-group and per-gene basis. Group relationships were illustrated in the 3D-PCA (Figure 2) which succinctly captures two major trends in the expression data. Namely, the clear sex- and age-related differences in expression shown by the discrete spatial separation of groups visualized in these parameters. Data from females follow a generally flat linear trajectory while results from males show conspicuous divergence from females during adulthood followed by convergence at late age. This pattern of late male convergence with female data is consistent with previous reports of increasingly "feminized" liver expression patterns in older rats [18]. Such a large divergence between males and females in the composite data suggests a substantial proportion of the 3,770 individual genes influencing this clear, sex-specific trend may be related to sex steroid biosynthesis pathways. In fact, upon analyzing the component or factor loadings for the first three principal components (Figure 2), genes from each principal component exhibited a unique characteristic. Principal component 1 (PC1), when ranked by factor loadings, consisted of genes exhibiting high female expression and low male expression (female-specific top genes: Cyp2c12 (cytochrome P450, family 2, subfamily c, polypeptide 12); Akr1b8 (aldo-keto reductase family 1 member b8); Sult2a1 (sulfotransferase family 2a member 1); Prlr (prolactin receptor)). PC2-ranked genes identified male-specific expression with concurrent low female expression (male-specific top genes: Mup5 (major urinary protein 5); Cyp2c13; Pgcl1/Pgcl3/Pgcl5 (alpha-2u globulins 1, 3, and 5); Cyp2c11). Whereas PC3-ranked genes included genes that exhibited large-fold temporal differences, yet were positively correlated between sexes (sex-conserved top genes: Cyp17a1; A2m (alpha-2-macroglobulin); Col1a1 (collagen, type I, alpha 1); Phgdh (3-phosphoglycerate dehydrogenase). The top 100 genes ranked by PC1 were analyzed for gene ontology representation (ArrayTrack's Gene Ontology For Functional Analysis, GOFFA) with "regulation of hormone levels", "steroid metabolic process", and "hormone metabolic process" as the top three GO terms which would support sex steroid hormone biosynthesis pathways explaining the biggest sex differences in PC1.

Linear continuity between neighboring age groups within each sex was maintained in the 3D-PCA, (e.g., 6 week animal data spatially appear between 5 week and 8 week animals for both males and females). Additionally, 3D-PCA permits the viewing of individual animal data as they contribute to the average or composite expression for one sex and age group. The relatively tight clustering observed among animals (biological replicates) of the same sex and age group illustrates the degree of reproducibility in sex- and age-related expression profiles. In fact, correlation coefficients (R) varied from 0.962 to 0.993 within a group when using DEGs, demonstrating high biological similarity. Subsequently, a k-means clustering method was used to bin individual genes into groups according to their temporal profiles (Figure 3). Such sorting algorithms establish the presence and prevalence of various patterns, thus suggesting global mechanisms of transcriptional control that can be assessed in the context of biological necessity or functionality in the liver. These clustered temporal profiles gain more interpretive potential when correlated with gross biological trends (such as the growth curves of animal body or liver weight) or developmental windows (sexual and physical maturation). The k-means clusters can then be associated with various developmental stages such as acute growth, puberty, early adulthood, and adult aging. For example, the transition from perinatal to pubertal control of growth and differentiation is believed to be directed in part by the switch from, Igf2 (insulin-like growth factor 2) expression to predominantly Igf-1 expression in the liver [36] during early development. These gene expression changes are illustrated in the different patterns shown in cluster 2 (Figure 3) for Igf-2 versus clusters 8 and 12 for Igf-1. Igf-2 exhibits high expression at 2 weeks (100- to 500-fold change above average for female and male, respectively) followed by relatively low and constant expression at the remaining age groups. Igf-1, however, shows low expression at 2 weeks followed by peak expression between 8 and 21 weeks in both sexes (Figure 5E). Another example, Phgdh, ranked high in the principal component 3 (PC3) factor loadings which exhibited sex-conserved, age-specific expression profiles. In this case, Phgdh fell into clusters 22 and 29 (male and female, respectively), demonstrating transient up-regulation between 5 and 6 weeks with declining expression in subsequent age groups. This expression pattern is consistent with the known association of Phgdh with highly replicating cells as the first and rate-limiting step in *de novo *biosynthesis of serine [37], which also plays a role as nucleotide precursor in regenerating liver tissue and hepatomas [38]. Thus Phgdh's transient up-regulation during early liver growth phase (5-6 weeks) is consistent with this physiological role. These patterns illustrate the temporal relationships between their expression and roles in liver development.

Several k-means clusters exhibit patterns of up-regulation with age. Clusters 6, 14 and 23 (Figure 3) in particular show an upward trend between 52 and 104 weeks, suggesting a group of genes important in the adult aging process. Examination of these genes using Ingenuity Pathway Analysis suggests many may be involved in compensatory mechanisms adapting to higher levels of liver injury, DNA damage or loss of function. For example, Mgmt (O6-methylguanine DNA methyltransferase) is a DNA damage repair enzyme which acts to directly reverse alkylation adducts in DNA [39]. Mgmt was found in clusters (clusters 11 and 23, male and female, respectively) which exhibit a trend toward of increasing expression in late adulthood (between 21 weeks and 104 weeks) (Figure 5D) which is consistent with previous reports associating Mgmt with aging phenotype [40]. Likewise, Por (P450 oxidoreductase) is a member of the "up-late" clusters. Por's microarray expression patterns were further verified by Taqman quantitative real time PCR (qRTPCR) (Figure 6). Interestingly, Por, along with cytochrome b5 [41], is a key electron donor for microsomal cytochrome P450 monooxygenases (CYP enzymes) [42], many of which function as Phase 1 metabolizing enzymes during xenobiotic metabolism. Por essentially acts to reload CYP enzymes in their monooxygenase activities. Por's reductase activities also target a number of other oxygenase enzymes involved in sterol and cholesterol biosynthesis, heme degradation to bilirubin, and fatty acid metabolism [42]. Thus, Por's consistent up-regulation from 52 weeks to 104 weeks could be explained by increased demand for CYP enzyme reloading due to accumulated xenobiotic exposure or fatty acid accumulation as a function of the adult aging process.

Other genes associated with "up-late" clusters (k-means clusters 1, 6, 9, 14, 23) included several oxidative stress related genes including Ephx1 (epoxide hydrolase 1), Fmo1, Fmo5 (flavin containing monooxygenase 1 and 5) and Hmox1 (heme oxygenase 1). Hmox1 is an important antioxidant protein expressed in response to oxidative stress [43], and thus its continuous increase in expression in older animals suggests a compensatory mechanism specifically to address increased levels of reactive oxygen species in the liver.

Expression level is not always an indicator of protein activity. For example, a previous report showed changes in male rat hepatic Cyp2e1 activity between 3 and 18 months of age despite the relative stasis of mRNA and protein levels [44]. However, examination of Cyp2e1 gene expression on a finer scale, as was done in this study, revealed sex-conserved spikes in young animals (between 2 weeks and 8 weeks) followed by greater than 2-fold differences in expression level between sexes at 21 weeks (Figure 7). Therefore, care must be taken in interpreting changes in mRNA expression levels on their own. This broader examination using more time points during the life cycle may prevent over-interpretation of expression data captured in studies of smaller scope.

Male rodents are preferred in many studies evaluating toxicological or pharmacologic response *in vivo *due to adult female variability in response during estrous cycle [45]. However, the current study using unsynchronized females is a crucial starting point to evaluating sex differences in liver expression over the larger scale of the life cycle. The number of replicates per age group (5 per age group) and relative consistency across multiple adult age groups increases our confidence in the sex-specific differences. Whereas Mori et al. [17] were limited to comparisons of relatively young (32 weeks) and old males (84 weeks), a full life-cycle analysis in both sexes provides comprehensive data on the temporal dynamics of gene expression and evaluates how well correlated male and female data are throughout the life cycle. If and when male and female data do not comport with each other, these sex-differences may inform our assessments of clinical (human) response which may influence the sex and age of rodent models used for pre-clinical testing. Wauthier et al [22] provide comprehensive lists of male- and female-specific liver gene expression in approximately 13 week old rats. A comparison of their top 100 male- and female-specific genes with the top 100 male/female-specific genes from the 15 week age group of the current data set was performed. An overlap of 73% (46/63, matched by Agilent probe ID) and 61% (43/71) for male- and female-specific expressed genes, respectively, were identified, suggesting a good degree of overlap of the most sex-specific genes. Furthermore, Lee et al. [14] complemented their male data with a meta-analysis of sex-specific genes from a separate study [46] and showed age-related changes in xenobiotic metabolism genes in the course of 6, 11, 18 and 24 month old rats. However, because the male and female data were not from the same study and the time and sex analyses were evaluated separately, inter-study confounding factors can not be adequately controlled for. Thus, analysis of sex- and age-related changes between animals within the same in-life study, as reported here, removes any uncertainties with regard to such confounding factors and allows for more robust comparisons.

Efforts have been made using differing metrics (comparing times of weaning, sexual maturity, skeletal maturity, etc.) to align the life cycle of normal human development and maturation with that of the rat [35], illustrating the compressed timescale that exists in rodent models. Properly extrapolating information about specific windows of development or aging from rodents to humans is therefore only approximate; however, the comprehensive gene expression profiles throughout the life cycle of the rat reported here may help further refine the life-cycle alignment of the rat model to human.

The FDA has assembled a listing of valid genomic biomarkers in the context of approved drug labels that are used in a variety of specific uses, including evaluating clinical response and differentiation; risk identification; dose selection guidance; susceptibility, resistance and differential disease diagnosis; and polymorphic drug targets [47]. Of the 20 gene-specific biomarkers on the FDA list, 13 of the genes were present on the Agilent arrays and 8 of them were differentially expressed by age or sex. Of these 8 genes, CYP2C9 (*r*Cyp2c11), CYP2D6 (*r*Cyp2d4), and EGFR (epidermal growth factor receptor), exhibited notable sex-differences in gene expression patterns in this study. Cyp2c11 showed much greater (200-250 fold difference) male-specific expression from 8 to 52 weeks of age. Both Cyp2d4 and Egfr showed ~1.5 and 2-fold higher expression, respectively, in males from 15 to 52 weeks. The relative expression of cytochrome P450 enzymes may have sex- and age-specific implications for the liver; however, Egfr was validated as a biomarker for cancer in non-hepatic tissues. Two other of the 9 DEGs, Dpyd (dihydropyrimidine dehydrogenase) and Nat1 (N-acetyltransferase 1), exhibited sex-conserved repression (~-2-fold) at early ages (2 and 5 weeks) followed by minimal changes at later ages. The remainder of the validated biomarkers that were differentially expressed during the rat life cycle showed few or minimal changes in expression that were very close to the screening criteria threshold. Thus, the expression levels of these biomarkers vary through the life-cycle of the rat model and this may have implications for their appropriate use.

Circadian rhythms have been shown to influence the expression of xenobiotic metabolism genes during the 24-hr cycle [48]. There are also reports that changes in circadian control during the aging process may, in addition, impact xenobiotic metabolism capacity during the life cycle [49]. Master circadian regulators Clock and Arntl/Bmal1 (aryl hydrocarbon receptor nuclear translocator-like) control the expression of the PARbZip family of transcription factors which is comprised of three members: Dbp (albumin site D-binding protein), Tef (thyrotroph embryonic factor), and Hlf (hepatocyte leukemia factor). In turn, these PARbZip transcription factors have been shown to regulate several enzymes and regulators involved in detoxification and drug metabolism, such as cytochrome P450 enzymes, carboxylesterases and constitutive androstane receptor [50]. The genes for two of these transcription factors, Dbp and Tef, were found to be differentially expressed throughout the life cycle in an age- and sex-specific manner (Figure 5A) and also to be highly correlated between the sexes (Pearson, R = 0.94). Furthermore, humans exhibit sexually dimorphic patterns of suprachiasmatic nucleus (SCN) neuron deterioration, with women showing no change throughout adulthood while men exhibit complex and fluctuating patterns with increasing age [51]. It is interesting to note that the pattern of SCN-controlled clock genes, Dbp, Tef, and Hlf (Hlf's 1.3-fold change did not meet criteria for inclusion in list of DEGs) display a conspicuous correlation with these reports (Figure 5A). Namely, while female expression remains consistent between 52 weeks and 104 weeks, males exhibit comparable levels of expression to female at 52 and 104 weeks but a noticeable decrease at 78 weeks. The microarray expression profiles of Dbp and Arntl/Bmal1 were further verified by qRTPCR (Figure 6). Based upon the known common pathway (Dbp and Tef controlled by the SCN) and life cycle expression data exhibiting correlated and sex-specific patterns, these data suggest a shared regulatory mechanism between the PARzip family members' expression and the sex-specific pattern of SCN neuronal deterioration. This further supports the concept that the circadian clock may influence life cycle "clocks" and thus influence downstream xenobiotic metabolism capacity.

The major mechanisms controlling sex differences in liver gene expression have been well-characterized [52] and are dictated by the distinct patterns of continuous vs. pulsatile plasma growth hormone (GH) secretion in females and males, respectively [53]. These temporal differences in plasma GH levels influence the activity of transcription factors Stat5b (signal transducer and activator of transcription 5b), Hnf4α (hepatic nuclear factor 4, alpha), and Stat5a (signal transducer and activator of transcription 5a), which then differentially activate sex-specific cytochrome P450s (CYPs) such as Cyp2c family members. Several Cyp2c family members analyzed in this study exhibited clear and conspicuous differences in expression, including Cyp2c7, Cyp2c11, Cyp2c13, Cyp2c12, Cyp2c79, and Cyp2c22. Previous studies of rat liver gene expression have also shown age-related changes in xenobiotic metabolism genes, although only male rats were examined, [17] and included Cyp2c11 and Cyp3a2. Both of these genes were represented on the Agilent array used in this study and showed comparable changes in gene expression at similar age groups along with related family members (Cyp2c13 and Cyp3a23/3a1, Cyp3a9 and Cyp3a18). A number of xenobiotic metabolism related receptors of interest were present on the array but were either not differentially expressed (pregnane X receptor, Nr1i2; constitutive androstane receptor, Nr1i3; aryl hydrocarbon receptor, Ahr; peroxisome proliferator-activated receptor alpha and gamma, Ppara/Pparg; retinoid x receptor alpha and beta, Rxra/Rxrb) or minimally passed threshold criteria for differential expression (retinoid X receptor, Rxrg). Cyp1a2, a notable xenobiotic and drug metabolizer and known target of Ahr, exhibited roughly parallel expression between sexes with maximum relative expression (~1.5 fold) peaking around 6 to 8 weeks followed by declining expression with age. Of the 34 phase I and II metabolizing genes found to be changed with age in male animals by Mori et al. [17], 27 were present in this study's list of DEGs and showed comparable expression changes across corresponding time scales. However, it is important to note that the female patterns differed remarkably from males in over 50% of those genes, raising uncertainties regarding how well data from male animals reflect the female response. Data for Cyp2c11 and Cyp3a23/3a1 are shown in Figure 7.

It has also been shown that GH regulates Cux2 (cut-like homeobox 2) which has been suggested [54] to act as a suppressor of male-specific gene expression. Laz et al. [54] employed *in silico *methods to predict binding sites of Cux2 suggesting target proximal genes for repression. In the current data set, when life cycle profiles for Cux2 were compared to the 16 suggested target genes, a clear sex-specific repressive relationship was observed with one of the genes, Cyp4a8 (R = -0.699 in males; Figure 5B). Cux2 showed approximately 12-fold expression ratio difference between males and females from puberty through early adulthood, while Cyp4a8 showed approximately 11-fold sex difference (male/female) in expression level. These comparative life cycle expression data provide further evidence of Cux2's role in sex-specific repression of Cyp4a8. Cyp4a8 is also a target of PPARα (peroxisome proliferator activated receptor alpha) signaling [55] and its expression has been suggested to explain sex and strain differences in the susceptibility to hypertension and target organ damage [56]. Thus these data suggest integrated sex-specific control of genes like Cyp4a8 that are also involved in xenobiotic receptor mediated responses.

To discover further large-scale patterns of sex-specific life cycle gene expression, the data were analyzed on a per-age group basis assessing the number of instances where the expression difference of a given gene between the sexes was at least 2-fold, with the condition that the data at that individual age group first met the screening criteria of p < 0.05 and great than or equal to 1.5 relative fold-change (Figure 4). The periods of the life cycle in which one sex predominates over the other in expression level appear to be non-random and appear at discrete times of the life cycle. For example, female predominant expression prevails during or soon after sexual maturation and into early adulthood (8 and 15 weeks) whereas male predominant expression appears to peak between 21 and 78 weeks of age. Interpreting this result in light of the 3D-PCA analysis, the data suggest that differences between males and females are observed immediately at 2 weeks, with more noticeable sex-divergence at approximately 6 weeks (Figure 2) which extended through sexual maturation. This sex-divergence seems to be due at first to genes being expressed at higher levels in females than males (Figure 4), followed by a transition where male expression is higher than females throughout the majority of adulthood. For example, Stat3 exhibits transient, female-specific predominance at 15 weeks while Cdh17 (cadherin 17) shows clear male-specific expression between 8 and 104 weeks (Figure 5C). Only one clear example, Cyp3a23/3a1 (Figure 7), was found among the 122 xenobiotic metabolism genes that exhibited both the female predominant expression at 15 weeks and male predominant expression at 52 weeks, suggesting that the sex-specific predominance of expression observed in Figure 4 primarily occurs in different sets of genes. The large-scale patterns observed in the global analyses (Figures 2 and 4) are also observed in individual gene profiles (Figure 7), including several cytochrome P450 family members in addition to other xenobiotic metabolizing proteins (Table 1).

## Conclusions

This study comprises one of the most comprehensive data sets to date for rodent liver gene expression with regards to sex, life span, biological replication, and genome coverage. A broad description of the dataset in the context of the normal physiology of the rat life cycle suggests important implications for toxicological susceptibility as related to xenobiotic metabolism, DNA damage repair and oxidative stress response. Namely, principal component analysis identified and ranked genes which exhibited the greatest sex-specific and sex-conserved differential expression. Furthermore, specific periods of the life cycle were identified that display a higher number of genes with sex-specific expression which included putative toxicological susceptibility related genes (e.g., Cyp2d4, Cyp3a23/3a1, Gstm1, Slc22a8). Cluster analyses, in combination with functional annotation analysis, identified gene clusters that positively correlated with adult aging which also included several susceptibility related genes (i.e., Por, Mgmt). Lastly, life cycle expression data provided experimental support for gene regulatory relationships previously based upon *in silico *predictions alone (i.e., Cux2, Cyp4a8).

The ability to make informed regulatory decisions about the impact of drugs and other chemicals on the liver is dependent upon a growing understanding of liver biology and more specifically the expression profiles of the molecular components which play a role in the pharmacokinetics and pharmacodynamics in liver cells. This large-scale study captures comprehensive baseline hepatic gene expression profiles during the life cycle of male and female rats which should prove to be an increasingly valuable resource for toxicogenomics-related research and ongoing evaluations of pre-clinical and clinical biomarkers of toxicity and efficacy.

## Methods

### Animal Study

Male and female (unsynchronized) Fisher 344 rats obtained from NCTR's animal breeding colony were fed the NIH-31 diet (*ad libitum*) and housed under AAALAC-approved conditions with a 12-hr light/dark cycle (0600 - 1800). Rats were housed two per cage in standard polycarbonate cages with hardwood chip bedding maintained at 23 degrees C with a relative humidity of ~50%. Animals were sacrificed at 2, 5, 6, 8, 15, 21 weeks (male and female n = 6 per age group), 52 weeks (males n = 10, female n = 10), 78 weeks (male n = 8, female n = 8) and 104 weeks (male n = 6, female n = 16) of age. Additional animals were included in the 52, 78, and 104 week time points to compensate for anticipated loss of animals during the course of the experiment. These numbers were based upon natural survival curves of these animals. Rats were treated according to the NCTR Institutional Animal Care and Use Committee guidelines.

### Necropsy

Animals were sacrificed at the same circadian time (between 0900 and 1100) for each time point and euthanized by carbon dioxide asphyxiation. Body weights were recorded and right lateral lobe sections of liver to be used for gene expression studies were stored in RNA*later *(Ambion, Foster City, CA) for 24 hr at room temperature followed by storage at -20 degrees C. Sections were collected at 52, 78 and 104 weeks for histological examination and placed in 10% neutral buffered formalin. Additional tissues including kidney, brain, pancreas, heart, lungs, muscle, uterus and testes were also collected in a similar manner.

### RNA Isolation

Total RNA was isolated from approximately 30 mg liver sections using Qiagen RNeasy Mini Kit (Qiagen Inc., Valencia, CA) according to manufacturer's protocol. The yield of the extracted RNA was determined spectrophotometrically by measuring the optical density at 260 nm (Nanodrop-1000, Thermo Scientific, Wilmington, DE). The purity and quality of extracted RNA were evaluated using the RNA 6000 LabChip and Agilent 2100 Bioanalyzer (Agilent Technologies, Palo Alto, CA). RNA samples with RNA integrity numbers (RINs) greater than 8.0 were used for microarray and real time PCR experiments with an average of 8.7 for all samples.

### Microarray Experiments

Gene expression studies (n = 5 per group) were completed using single color (Cy3) Agilent Whole Rat Genome 4 × 44k microarrays, which contain 4 identical arrays per slide, and reagents according to manufacturer's protocols (Agilent Technologies, Santa Clara, CA) for cRNA labeling and hybridization using 500 nanograms of total RNA. An Agilent one-color spike-in kit was used as a positive control and to monitor labeling efficiency across all experiments. A universal rat reference (URR) RNA (Stratagene, Agilent Technologies) was labeled and incorporated into the array design to control for batch/day effects during data processing. In total, six URR hybridized arrays were included in the study. Pair-wise Pearson's correlations between un-normalized individual URR array intensity values (F532-median) ranged from R = 0.968 to 0.997. Samples were randomized by sex and age such that biological replicates of the same group were not hybridized to the same slide. Arrays were scanned for fluorescent signal intensities using a GenePix 4000B microarray scanner (Molecular Devices, Union City, CA). Images were analyzed for feature and background intensities using GenePix Pro 6.0 software (Molecular Devices). Full microarray data are available at Gene Expression Omnibus with accession GSE21335 [57].

### Microarray Data Analysis

Microarray intensity data were uploaded to the ArrayTrack database [26] and normalized using 75^th^-percentile scaling without background subtraction. Statistical analyses were performed on normalized intensity values using a two-way ANOVA (sex and time-point) in ArrayTrack. Relative fold-change values were calculated for data from each sex and time point. These relative fold-changes (individual values divided by the averaged expression from both males and females) were calculated on a per spot/feature basis. The MicroArray Quality Control (MAQC) Consortium [58] suggests the use of a fold-change cut off along with a non-stringent p-value cut off as a baseline practice to improve reproducibility in microarray data processing. Therefore, filtering criteria, consisting of a p-value < 0.05 and an absolute fold-change value of 1.5 or greater at any time point, were used to define an initial set of differentially expressed genes. Applying these criteria to the 41,897 features on the Agilent microarrays resulted in 7,951 array features (consisting of 3,770 unique Entrez Gene IDs) being designated as differentially expressed. The complete data set with annotations, fold-changes and p-values, k-means clusters, and 3D-PCA loadings data is available in Additional File 1, Table S1. For brevity and consistency, genes are referenced by their official gene symbol as defined by NCBI. Three-dimensional principal component analysis (3D-PCA) was performed on normalized intensity values of the 7,951 differentially expressed features in ArrayTrack. K-means cluster analysis was also performed on the 7,951 differentially expressed features using JMP Genomics (SAS 9.2). The number of initial clusters chosen was 30 as this was the lowest number of clusters to allow a minimal correlation coefficient of R = 0.7 for any feature profile in its respective cluster. Functional annotation and pathway analysis of gene expression data was performed using Ingenuity Pathway Analysis (Ingenuity Systems, Redwood City, CA). Default settings for expression dataset analysis were used and results from Top Networks, Bio- and Tox- Functions and Canonical Pathways were queried to facilitate the biological interpretation of gene lists.

### Quantitative Real Time PCR Analysis

For each sample, 0.5 μg of total RNA was reverse transcribed by MultiScribe reverse transcriptase using random primers as described by the manufacturer (Applied Biosystems, High-Capacity Reverse Transcription Kit). The resultant cDNA (1.0 μl) was used as the template in a 20 μl Taqman Expression Assay PCR reaction (Applied Biosystems, Foster City, CA) for Cyp2d4 (Assay ID: Rn01504629_m1), Por (Rn00580820_m1), Arnl (Rn00577590_m1), Dbp (Rn00497539_m1) and Actb (Rn00667869_m1). Taqman PCR was conducted in MicroAmp Optical 96-well reaction plates (Applied Biosystems) on an ABI 7500 real-time PCR detection system. The gene expression level of each sample for each gene was standardized to the house-keeping gene, Actb, to control for differences in RNA loading, quality and cDNA synthesis using the ΔΔCt method. For graphing purposes, the relative expression levels were scaled such that the expression level of the mean expression for males and females was equal to one.

## Abbreviations

3D-PCA: three dimensional principal component analysis; AAALAC: Association for Assessment and Accreditation of Laboratory Animal Care; BER: base excision repair; Cyp: cytochrome P450; DEGs: differentially expressed genes; DILI: drug induced liver injury; F344: Fisher 344; FDA: Food and Drug Administration; GH: growth hormone; GO: Gene Ontology; GOFFA: Gene Ontology For Functional Analysis; IPA: Ingenuity Pathway Analysis; MMR: mismatch repair; NCBI: National Center for Biological Information; NCTR: National Center for Toxicological Research; NER: nucleotide excision repair; NTP: National Toxicology Program; PAR bZip: proline and acidic amino acid rich basic leucine zipper; PC: principal component; PPARa: peroxisome proliferator activated receptor alpha; qRTPCR: quantitative real time PCR; RIN: RNA integrity number; SCN: suprachiasmatic nucleus; SEM: standard error of the mean; URR: universal reference RNA.

## Competing interests

The authors declare that they have no competing interests.

## Authors' contributions

JCK carried out the microarray experiments, data processing, pathway analysis and drafted the manuscript. VGD participated in the study's conception, design and coordination of the in-life study. CLM participated in the in-life study and RNA isolation. WSB participated in the in-life study and microarray data processing. JCF conceived of the study, and participated in its design and coordination and helped to draft the manuscript. All authors read and approved the final manuscript.

## Supplementary Material

Additional file 1 **Supplementary Data Table S1**. A listing of all differentially expressed genes, annotations, relative fold change values, p-values, k-means clusters and principal component analysis loadings values. Open with MS Excel or comparable spreadsheet software. Click here for file
